# Mental health in the aged: prevalence, covariates and related neuroendocrine, cardiovascular and inflammatory factors of successful aging

**DOI:** 10.1186/1471-2288-10-36

**Published:** 2010-04-30

**Authors:** Maria E Lacruz, Rebecca T Emeny, Horst Bickel, Barbara Cramer, Alexander Kurz, Martin Bidlingmaier, Dorothea Huber, Günther Klug, Annette Peters, Karl H Ladwig

**Affiliations:** 1 Institut für Epidemiologie - Helmholtz Zentrum München, München, Germany; 2 Klinik und Poliklinik für Psychiatrie und Psychotherapie - Klinikum rechts der Isar, TU München, Germany; 3 Medizinische Klinik der Innenstadt - Ludwig-Maximilians-Universität München, München, Germany; 4 Klinik und Poliklinik für Psychosomatische Medizin und Psychotherapie - Klinikum rechts der Isar, TU München, Germany

## Abstract

**Background:**

Although aging is accompanied by diminished functioning, many elderly individuals preserve a sense of well-being. While the concept of "successful aging" has been popular for many decades, little is known about its psycho-physiologic and endocrine underpinnings. KORA-Age is a population-based, longitudinal study designed to determine the prevalence of successfully aged men and women between 65 and 94 years old in the MONICA/KORA Augsburg cohort of randomly selected inhabitants. Specifically, we aim to identify predictors of successful aging and to elucidate bio-psychosocial mechanisms that maintain mental health and successful adaptation despite adverse experiences of life and aging.

**Methods/Design:**

Components of successful aging were assessed in a telephone survey of 4,127 participants (2008-2009) enrolled in the MONICA/KORA cohort, on average, 13 years earlier. Psychosocial, somatic and behavioural predictors are used to determine factors that contribute to successful aging. An age-stratified random sub-sample (n = 1,079) participated in a personal interview where further psychological mechanisms that may underlie successful adaptation (resilience, social support, attachment) were examined. The interactions among neuroendocrine systems in the aging process are investigated by studying the cortisol/dehydroepiandrosterone-sulfate ratio, the level of insulin-like growth factor I, and oxytocin.

**Discussion:**

Longitudinal determinants of successful aging can be assessed based on a follow-up of an average of 13 years. A comprehensive analysis of biological as well as physio-psychological information provides a unique opportunity to investigate relevant outcomes such as resilience and frailty in the elderly population.

## Background

The elderly constitute the fastest growing segment in the population. Increasing age is often associated with worsening physical and mental health, decreasing functional ability and cognitive impairment [1]. Moreover, many older individuals face social isolation due to physical constrains. Nevertheless, an undefined portion of the older population achieves "successful aging" and thereby maintains a sufficient level of subjective well-being. These persons continue to have future plans, a desire to accomplish new goals, and they exhibit a sense of happiness and joy [2]. At least for these individuals, "...old age is not foremost a negative and problem-ridden phase of life" [3]. However, little is known about the sex-specific prevalence of successfully aged individuals in the general population [4], or whether this characterization is stable over time. Furthermore bio-psychological mechanisms of decline in the elderly are uncertain.

There is no unanimous definition of "successful aging". To date, it remains unclear whether the construct refers simply to the absence of disability or to the combination of specific positive attributes among others, well-being. A recent literature search identified 28 studies with 29 different definitions [5]. The Honolulu Aging Study [6] defined "exceptional survival" as survival to 85 years, without incidence of 6 major chronic diseases and without physical and cognitive impairment. Only 11% of 2,451 male participants investigated met the criteria.

However, successful aging has been also defined as more than the absence of disability and multi-morbidity. Even in the context of chronic physical impairment, aged individuals may experience successful aging, which points to a distinction between being chronically ill and regarding oneself as sick [7,8]. Accordingly, the Berlin Aging Study [3] revealed that the elderly take an average of three to eight different medicines and therefore may be chronically ill in the eyes of their physicians, but often do not regard themselves as sick. Self-perceived health is the most common definition of successful aging in the general population [8] and is also a highly significant predictor of mortality [9,10].

Rowe & Kahn [11] distinguished between "normal" and "successful" aging - the latter a combination of three components: a) absence or avoidance of disease and risk factors for disease, b) appropriate physical and cognitive functioning, and c) active engagement with life (including autonomy and social support). Although this is the most widely used approach in aging research - it fails to address the implications of the fact that a disease-free older age is unrealistic [8]. Apparently, the concept of successful aging rests on a continuum of achievement [8] rather than success or failure, and its definition requires a combination of biomedical and psychosocial approaches.

Specialised fields of study share only minimal agreement with the view of the elderly themselves. The Leiden85-plus Study [12] gained deeper insight into the perspective of the very old (above 85). For individuals over 85, successful aging was equivalent to well-being. Additionally, study participants expressed their sense of health as the maintenance of basic functions (i.e. vision, hearing, and mobility) and the absence of life-threatening diseases. They also perceived social functioning as being essential for well-being. Only 10% of all participants could be classified as having an "optimal state" of overall functioning reporting only minor physical disabilities, regular social activities, and good cognitive function and sense of well-being. However, almost half of the participants over 85 reported an optimal state of well-being despite limited functioning. Thus, successful aging may not be an objective measurement of physical function but rather a process of adaptation to physical and functional limitations.

There are four objectives in this study: 1) to determine the prevalence of different definitions of successful aging in a representative sample of elderly individuals in the MONICA/KORA study; 2) to identify predictors of successful aging; 3) to phenotype the mental health of the elderly; 4) to identify biomarkers of successful aging. These aims are briefly described below.

### Objective 1: Successful Aging Prevalence Study, n = 4,127

In the present investigation, different definitions of successful aging will be analysed (Table 1):

**Table 1:** Definitions of successful aging

Successful Aging Approach	Definition	References
Biomedical	Absence of multi-morbidity	[6]

Psycho-Cognitive	Absence of depression, absence of anxiety, no cognitive impairment, regular functioning, quality of life, resilience	[3,12,14,38]

Lay View Approach	Presence of well-being, good self perceived health	[8,12]

Combined Approach	Optimal functioning	[6,12]

The purpose of our analysis is to assess the age- and sex-related prevalence of participants above 65 years who meet the criteria of successful mental and cognitive aging based on a random sample of the population recruited between 1984 and 2001 [13]. In collaboration with consortium partners, we will also incorporate a biomedical, functional and laymen's approach of successful aging and will investigate the extent of associations between these different approaches.

Specific aims are:

• To assess the prevalence of successful aging according to the psycho-cognitive, biomedical, laymen's and combined approach.

• To investigate the inter-relation of different domains of successful aging

• To assess gender and age-related differences in the prevalence of successful aging

• To characterize participants who meet the psycho-cognitive criteria of successful aging, despite somatic and functional impairments (multi-morbidity and disability).

### Objective 2: Successful Aging Predictor Study, n = 4,127

Successful aging may not occur by chance. However, knowledge about predictors and sequential events that facilitate successful aging is sparse. The main objective is to elucidate the predictive power of a wide range of midlife psychosocial, somatic and behavioural factors that contribute to successful aging. Elderly participants with a good mental health as defined in Objective 1, will be compared to those with impaired mental health on the following variables, which were assessed in their midlife:

#### Socio-demographic and workplace features

• Education and social class

• Social network

• Work conditions

• Chronic psychological stress and job stress

#### Mental health and behavioural features

• Hyperactivity and/or hostile behaviour patterns (Type A/B Patterns)

• Depression and/or mental exhaustion and/or Type D behaviour

• Sleep disorders

#### Health behaviour and risk factors

• Cardiovascular risk factors, in particular diabetes, hypertension, hypercholesterolemia and hypertriglyceridemia

• Life style: physical activity, healthy nutrition, smoking

• Self-perceived health and/or somatic complaints

#### Cognitive impairment and risk factors

There is growing evidence that midlife cardiovascular risk factors are associated with vascular and even primary degenerative dementias, including Alzheimer's disease [14-18]. However, the relationship between risk factors and dementia involves a long exposure time. Accordingly, studies investigating this association need to encompass an observational period of several decades. Therefore, cohorts in whom relevant risk factors have been assessed already in midlife are uniquely informative. The MONICA/KORA database offers a unique opportunity to investigate the effects of vascular risk factors on the risk of cognitive impairment over several decades.

### Objective 3: Mental Health Phenotype Study, n = 1,079

The aim of this study is to investigate the influence of external object relations (social support) and internal object relations (attachment) on the development of late-life depression. The absence of major depression is among the strongest early predictors of successful aging [19]. Among the typical causes of late-life depression is the loss of family and friends. Furthermore, the inevitable transitions of this period, such as retirement or onset of a disease, may seriously impair an individual's social support network and can lead to depression. It is a clinically and empirically well known fact, however, that not every loss or impairment of social support leads to depression. Single case studies and clinical knowledge suggest that some aged people seem to be protected against depression by an inner world of good object representations, the result of secure attachment. This indicates the enormous importance of the inner world of object representations [20]. These unsystematic findings are not yet empirically supported.

The following hypothesis will be tested: Individuals with good social support and secure attachment have the lowest prevalence of late-life depression, followed by those with little social support but secure attachment, and finally the highest rates of depression will be observed in participants with little social support and no secure attachment.

The novel aspect of the study is the systematic investigation of social support and attachment for the development of late-life depression in a large sample of elderly participants.

### Objective 4: Successful Aging Biomarker Study, n = 1,079

The purpose of this study is to identify biomarkers that are individually, or in combination, associated with successful aging. With this aim, we will define quantitative traits of successful and unsuccessful aging (as characterised in Objective 1) in the random sub-sample of 1079 participants. We will analyse the circulating concentrations of pituitary and adrenal hormones at rest and will focus on cortisol and DHEA-S (dehydroepiandrosterone-sulfate) (adrenal activation), IGF-I (insulin-like growth factor) (somatotropic axis) and oxytocin (neurohypophysis). Only a few studies thus far have paid particular attention to the aged, and those investigating individuals above the age of 65 have mainly used a purely somatic definition of disease. Furthermore, most of the studies are "treatment oriented", i.e. those attempting to prove the efficacy of hormone (replacement) therapy. Within the concept of "successful aging" outlined above, and especially with respect to the difference between "being chronically ill" and "self-perceived health", no studies are presently available that attempt to define a "hormonal profile" potentially associated with successful aging.

In general, it is our hypothesis that overall "successful aging", well-being, indices of mental health, cognitive function and - as a consequence - overall health status are more appropriately reflected by ratios between stress hormones (cortisol) and protective hormones (DHEA-S, IGF-1, oxytocin). This approach may identify disturbances in the balance between the activation of the hypothalamic-pituitary-adrenal (HPA) axis and the ability of an individual to cope with such challenges.

## Methods/Design

### Recruitment of participants and time schedule

**Figure 1:**
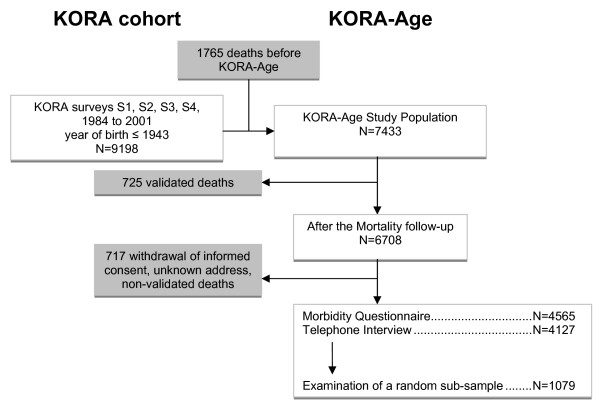
**Flow chart KORA-Age**.

The course of the KORA-Age project is depicted in Figure 1. Three study designs have been chosen to answer the questions presented above.

1) A mortality follow-up in 2008 of all 65 - 94 year old participants of the KORA cohort. N = 4,564.

2) A telephone interview of living participants from the KORA cohort older than 65. N = 4,127.

3) A medical examination of a sub-probe of the cohort 65 - 89 years old. N = 1,079

**Figure 2:**
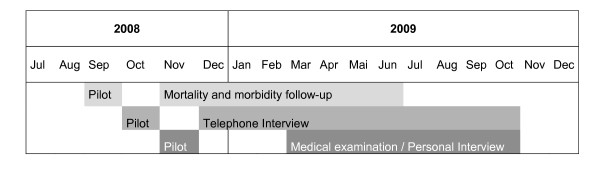
**Time schedule**.

These interviews and examination were carried out between September 2008 and November 2009 (see Figure 2).

KORA-Age invited all participants of the MONICA/KORA surveys (S1 to S4), who were older than 65 years at the end of December 2007, to partake. The surveys were carried out in 1984/85 (S1), 1989/90 (S2), 1994/95 (S3) and 1999/2001 (S4). All these surveys were based on representative samples of the population of Augsburg and two surrounding counties. Participants were 25 to 74 years old at the time of each survey. The participation rates ranged between 79% in S1 and 67% in S4 [13].

### Objective 1. Successful Aging Prevalence Study

The current research project is a part of the telephone interview survey and home or on-site visits which includes approximately 4,127 participants of the former MONICA/KORA surveys. The mental health module is a composite of instruments tailored to screen for important features of mental health indicators in participants in the age groups of 65 - 94 years.

• *WHO-Five Well-being Index *covers positive mood (good spirits, relaxation), vitality (being active and waking up fresh and rested), and general interests (being interested in things) [21,22]. Each of the five items is rated on a 6-point Likert scale from 0 (= not present) to 5 (= constantly present). The theoretical raw score ranges from 0 to 25 and is transformed into a scale from 0 (worst thinkable/possible well-being) to 100 (best thinkable/possible well-being). Thus, higher scores mean better well-being. A raw score below 13 indicates poor well-being and is an indication for testing for depression under ICD-10.

• *Euro-Qol (EQ-5D) *describes the health-related quality of life states consisting of five dimensions (mobility, self-care, usual activities, pain/discomfort, and anxiety/depression) each of which can be reported as one of three responses. The responses record three levels of severity (no problems/some or moderate problems/extreme problems) within a particular EQ-5D dimension, and yields a range of scores from 5 to 15 [9,10].

• *GDS-15 (Geriatric Depression Scale) *from Sheikh and Yesavage [23] evaluates depression. The short-form of the GDS (GDS-15) can give even patients with slight and medium cognitive limitations, a reliable indication of the existence of depressive symptomatology. The maximal score from GDS-15 is 15 points, where 0-5 indicates normal, 6-10 light to moderate depression, and 11-15 severe depression.

• *Generalised Anxiety Disorder scale (GAD-7)*: A recently developed screening instrument to assess generalised anxiety disorder [24] is available in a German version and consists of 7 items (4-point Likert scale). A cut-off point of 10 was identified that optimized sensitivity (89%) and specificity (82%).

• *Resilience*: We will apply the German version of the 11-item short form Resilience scale [25] to assess resilience as a protective personality factor. The German version was recently validated in an elderly sample from the general population [26].

The total interview time by the successful aging mental health requires approximately 7 to 13 min (out of 26 min. planned for the whole interview) depending on the cognitive status of participants.

• *Cognitive Status*: Participants will be screened by telephone using a short cognitive status test - the modified Telephone Interview for Cognitive Status (TICS-m) [27]. The TICS-m consists of 11 items that yield a total score from 0 to 50; lower scores are indicative of cognitive impairment. Time required to administer the test by telephone is only 6 to 8 minutes. A cut-off of ≤ 29 will be employed as indicative of cognitive impairment. The sensitivity of the TICS-m for the detection of dementia was estimated at 92% with a specificity of 100% [28].

### Objective 2. Successful Aging Predictor Study

All participants included in the Successful Aging Predictor Study are derived from the ongoing MONICA/KORA Augsburg Cohort Studies. Extensive and highly quality controlled data sets have been taken from the participants including anthropometric, laboratory measures, and psychosocial data sets. These data provide the unique opportunity to screen for a wide range of predictors for successful aging covering a life span of up to 25 years [13].

### Objective 3. Mental Health Phenotype Study

Depressive symptomatology will be assessed with the Geriatric Depression Scale (GDS-15). Social network will be assessed with the "Social Network Index". The scores of this index are between 1 and 4 [29]. The "social network index" is based on the "social network scale" ("Berkman-Index") of the Alameda-County-Study [30] and is detailed and reproduced in the WHO: MONICA Psychosocial Optional Study (MOPSY) [29]. Attachment will be measured with the Relation-Specific Attachment Scales for Adults (*Beziehungsspezifische Bindungsskalen für Erwachsene*: BBE). The BBE is a 14 items self-rating questionnaire to measure attachment styles of adults along the dimensions of "secure-fearful" and "dependent-independent" [31,32].

### Objective 4. Successful Aging Biomarker Study

To define a "neuroendocrine profile" potentially associated with successful aging, we have selected specific hormones of hypothalamic-pituitary-adrenal axis origin, which decline substantially with age and may be involved in the maintenance of cognitive function and mental health. We expect to observe age- and sex related differences in hormone concentrations. To allow identification of differences between the groups related to "successful aging", the population size was chosen based on these assumptions on hormone level variability (Table 2):

**Table 2:** Parameters and expected values (Mean ± 2SD)

Parameter	Age group	Males Mean (± 2SD)	Females Mean (±)	Unit	Reference
Cortisol	♂ 61-70 (n = 50)	12,9 (6,7-21,7)	-	μg/dl	Kit insert

DHEA-S	♂ 61-70 (n = 50) ♀ postmenopausal (n = 60)	136 (77-270)	55 (15-190)	μg/dl	Kit insert

IGF-I	66-70 (n = 22)	115 (66-198)	115 (66-198)	ng/ml	Elmlinger et al.[39]
		
	71-75 (n = 56)	107 (61-168)	107 (61-168)	ng/ml	
		
	>75 (n = 20)	94 (52-174)	94 (52-174)	ng/ml	

The respective data for oxytocin are not available yet. In a smaller study, Forsling et al. [33] reported mean morning concentrations of oxytocin around 4 - 1 pmol/l (mean ± SEM). Samples will be taken at the KORA Study centre and the pre-analytical phase will be organized according to the method specific requirements for each analytic. All hormone measurements will be done by validated methods established in the Endocrine Lab at the Ludwig Maximilian's University Clinic, Munich.

### Statistical analysis and power calculations

For descriptive statistical analysis, associations between categorical variables will be carried out with the chi-square test. Group differences in continuous variables will be analysed by the t-test (variables with 2 categories) or the F test (more than 2 categories). To test for trends of different definitions of successful aging (Objective 1) in 5-year age intervals, groups will be coded with their respective median values. Characteristics associated with increased successful aging (Objectives 2-4) will be identified by age-adjusted and by multivariable logistic regression analysis with stepwise variable selection. To account for the possibility of effect modifications, second-order interaction terms for all factors fulfilling the entry criterion will be included in the initial regression model. All analyses will be performed separately for men and women. Moreover, to assess age-adjusted sex differences in prevalence of various successful aging definitions, logistic regression models with age, the respective characteristic, sex and the interaction term characteristic*sex will be included in the model equation. We recently gained experience [34] with the *regression and classification tree *(CART) technique [35] which is particularly attractive in response variables offering a broad range. CART is a tree-based approach with a sequence of tests to assess a significant difference in a response variable (i.e. mean well being) by an explanatory variable (i.e. socio-demographic, psycho-diagnostic and biological variables). In each step, the explanatory variable with the lowest p value drawn from the test is chosen as a "node". With this minimum p value approach, the study population is divided into subsequent different dichotomous subclasses. This multivariate procedure allows the identification of different subclasses of the study population with respect to an outcome variable. In the final step, CART technique will offer sub-classes of participants with different mean well-being values. For all statistical analysis, a p value < 0.05 will be considered significant. Power calculations were made for assumptions and estimated prevalences of response variables.

### Ethical approval

Participants will be informed about research procedures and will be asked to sign an informed consent. The study protocol was submitted and approved by the Ethical Committee of the Bavarian medical association (Ethik-Kommission Nr. 08064).

## Discussion and Conclusion

This study protocol illustrates a collaborative work useful for researchers interested in innovative and realistic investigation, which is aimed at understanding the psycho-bio-medical-social characteristics of successful aging in a population-based sample. A discussion of specific strengths and limitations of this study follows below.

The first and obvious strength of this study is its feasibility, because the study design is based on the ongoing MONICA/KORA studies, which began in 1984 in Augsburg, Germany. All relevant methodological and logistical features of the study were successfully tested in previous studies, when, for example, a very low loss to follow-up rate was achieved [13]. Specifically, a response rate of 76% for the questionnaire and 69% for the telephone interview was achieved in KORA-Age.

Another strong aspect of the design of this study is the large number of measurements. A great variety of psychological tests and biomarkers are used, making it possible to analyze the role of potential mediating variables as predictors of successful aging as well as the development of multiple symptoms over time.

An additional advantage of this study concerns the large sample size, making it easy to draw firm conclusions about prevalence rates, and analytic results.

This is a non-intervention, prospective cohort study with the limitations inherent to non-experimental research. The possibility of bias and residual confounding can never be entirely eliminated, and the ability to infer causation is correspondingly limited [36]. Nonetheless, our current, improved insight into potential sources of bias and confounding as well as the use of refined statistical and epidemiologic methodology helps to estimate the impact of bias and residual confounding. This difficulty may however, remain unresolved when all that exists is a weak association. In practical terms, a point in the gradient of declining relative risk must be reached where the amount of bias and residual confounding becomes so small that it cannot realistically be ruled out [37].

In conclusion, it is clear that society is faced with an ever growing elderly population with increasing medical demands. This study aims to contribute to the evidence-based research that may promote individual well-being in the elderly.

## Abbreviations

(DHEA-S): dehydroepiandrosterone-sulfate; (IGF-I): insulin-like growth factor; (EQ-5D): EuroQol Instrument; (GDS-15): Geriatric Depression Scale; (GAD-7): Generalised Anxiety Disorder scale; (TICS-m): modified Telephone Interview for Cognitive Status; (MOPSY): MONICA Psychosocial Optional Study; Relation-specific Attachment Scales for Adults (Beziehungsspezifische Bindungsskalen für Erwachsene: BBE); (CART): classification and regression tree.

## Competing interests

The authors declare that they have no competing interests.

## Authors' contributions

MEL and RTE will carry out analysis and interpretation of data, and drafted the manuscript. KHL, BH, CB and AK planned the 1^st ^objective. KHL planned the 2^nd ^objective, initiated the study, formulated the research grant and drafted the manuscript. KHL, DH and GK planned the 3^rd ^objective. KHL and MB planned the 4^th ^objective. AP is coordinator of KORA-Age: All authors read and approved the final manuscript.

## Pre-publication history

The pre-publication history for this paper can be accessed here:

http://www.biomedcentral.com/1471-2288/10/36/prepub
